# Effect of Hashimoto’s thyroiditis on the dual-energy CT quantitative parameters and performance in diagnosing metastatic cervical lymph nodes in patients with papillary thyroid cancer

**DOI:** 10.1186/s40644-024-00655-1

**Published:** 2024-01-18

**Authors:** Di Geng, Yan Zhou, Ting Shang, Guo-Yi Su, Shu-shen Lin, Yan Si, Fei-Yun Wu, Xiao-Quan Xu

**Affiliations:** 1 Department of Radiology, The First Affiliated Hospital of Nanjing Medical University, No. 300, Guangzhou Road, Nanjing, PR China; 2 Department of Radiology, Affiliated Hospital of Integrated Traditional Chinese and Western Medicine of Nanjing University of Chinese Medicine, Nanjing, China; 3 Siemens Healthineers Ltd, Shanghai, China; 4 Department of Thyroid Surgery, The First Affiliated Hospital of Nanjing Medical University, Nanjing, China

**Keywords:** Papillary thyroid cancer, Thyroiditis, Lymphatic metastasis, Multidetector computed tomography

## Abstract

**Background:**

To evaluate the effect of Hashimoto’s thyroiditis (HT) on dual-energy computed tomography (DECT) quantitative parameters of cervical lymph nodes (LNs) in patients with papillary thyroid cancer (PTC), and its effect on the diagnostic performance and threshold of DECT in preoperatively identifying metastatic cervical LNs.

**Methods:**

A total of 479 LNs from 233 PTC patients were classified into four groups: HT+/LN+, HT+/LN−, HT−/LN + and HT−/LN − group. DECT quantitative parameters including iodine concentration (IC), normalized IC (NIC), effective atomic number (Z_eff_), and slope of the spectral Hounsfield unit curve (λ_HU_) in the arterial phase (AP) and venous phase were compared. Receiver operating characteristic curve analyses were performed to evaluate DECT parameters’ diagnostic performance in differentiating metastatic from nonmetastatic LNs in the HT − and HT + groups.

**Results:**

The HT+/LN + group exhibited lower values of DECT parameters than the HT−/LN + group (all *p* < 0.05). Conversely, the HT+/LN − group exhibited higher values of DECT parameters than the HT−/LN − group (all *p* < 0.05). In the HT + group, if an AP-IC of 1.850 mg/mL was used as the threshold value, then the optimal diagnostic performance (area under the curve, 0.757; sensitivity, 69.4%; specificity, 71.0%) could be obtained. The optimal threshold value of AP-IC in the HT − group was 2.050 mg/mL. In contrast, in the HT − group, AP-NIC demonstrated the highest area under the curve of 0.988, when an optimal threshold of 0.243 was used. The optimal threshold value of AP-NIC was 0.188 in the HT + group.

**Conclusions:**

HT affected DECT quantitative parameters of LNs and subsequent the diagnostic thresholds. When using DECT to diagnose metastatic LNs in patients with PTC, whether HT is coexistent should be clarified considering the different diagnostic thresholds.

## Introduction

Papillary thyroid cancer (PTC) accounts for up to 90% of the malignant tumors in the thyroid [1]. Cervical lymph node (LN) metastasis is highly associated with a poor prognosis of PTC [2]. Therefore, accurate preoperative identification of metastatic LNs is important for establishing the individual treatment plan for patients with PTC. Ultrasonography (US) is usually first suggested for evaluating the status of cervical LNs in patients with PTC in clinical practice [2]. However, US is operator dependent, and it is limited in the evaluation of central LNs because of the gas interference in the trachea and esophagus and the shield of the sternum and clavicle [3, 4]. Although administration of iodine-containing contrast can potentially suppress thyroidal radioactive iodine uptake and subsequently postpone radioiodine therapy [5], contrast-enhanced computed tomography (CT) can provide more detailed anatomic information, and previous studies have reported that CT combined with US can improve the sensitivity in diagnosing metastatic LNs [4, 6]. However, it is typical to assess the morphology-based imaging features of these two modalities for subsequent differential diagnosis, which is subjective. An objective approach that can provide quantitative biological information is needed in the clinical setting.

Dual-energy CT (DECT) is an advanced imaging technique that allows high- and low-energy image acquisitions using two different X-ray tube voltages, subsequently generating a variety of material decomposition images [7]. By reconstructing multiparameter images, DECT could provide more quantitative information by calculating parameters including iodine concentration (IC), effective atomic number (Z_eff_) and slope of the spectral Hounsfield unit curve (λ_HU_), which offers a noninvasive alternative to tissue characterization [8]. Previously, Liu X et al. reported that λ_HU_, normalized IC (NIC), and normalized Z_eff_ derived from DECT of cervical metastatic LNs were higher than those of nonmetastatic LNs [9]. DECT may be a promising approach for diagnosing the metastatic LNs in patients with PTC [9].

Recently, the coexistence of Hashimoto’s thyroiditis (HT) in patients with PTC has attracted increasing attention [10]. Moreover, previous studies have proved that the incidence of PTC is higher in patients with HT [11, 12]. HT is characterized by the lymphocytic infiltration caused by relevant autoantibodies [13]. A similar immune response can also occur in the thyroid-draining cervical LNs, subsequently leading to reactive lymphadenopathy. Concerning location, enlarged infrathyroidal and/or pretracheal reactive LNs are commonly observed in patients with HT [14, 15]. Meanwhile, in clinical practice, we also find that reactive hyperplastic LNs in patients with HT also often display a marked enhancement on CT images, which increases the difficulty in differentiating metastatic from nonmetastatic LNs. However, to the best of our knowledge, to date, there is still a dearth of studies focusing on the effect of HT on the DECT parameters and subsequent performance or threshold in diagnosing metastatic LNs.

Therefore, the purpose of our study was to: (1) examine the effect of HT on the DECT-derived quantitative parameters of cervical metastatic and nonmetastatic LNs and (2) evaluate the effect of HT on the performance and threshold of DECT-derived quantitative parameters in preoperatively diagnosing metastatic cervical LNs in patients with PTC.

## Materials and methods

### Study population

This retrospective study was approved by the institutional ethics committee (2022-SRFA-035), and the requirement for written informed consent was waived. Between June 2017 and June 2022, a total of 2,263 consecutive patients with suspected PTC underwent preoperative DECT scans for pretreatment evaluation. We retrospectively included the study population according to the following criteria: (1) confirmation of PTC by histopathology, (2) cervical LN dissection was performed and the pathological result was obtained, (3) DECT scans were examined within 1 month before surgery and lymph nodes with maximum short axis diameters ≥ 5 mm were found on images, (4) no history of radiotherapy or chemotherapy before surgery, (5) no history of other malignancies, and (6) adequate DECT image quality for subsequent analyses. Diagnosis of coexistent HT was established according to the method proposed in a previous study [16]. Specifically, PTC patients were considered to have coexistent HT if “Hashimoto’s thyroiditis,” “lymphocytic thyroiditis,” or “chronic lymphocytic thyroiditis” was reported in postoperative pathology. Finally, 233 patients (including 124 patients with HT) were included in our study. All patients enrolled were euthyroid. In addition, urinary iodine concentrations were measured in 87 of 233 patients. The median urinary iodine level was 235.8 µg/L (reference range, 100.0–300.0 µg/L). Detailed patient characteristics are presented in Table 1.

**Table 1:** Demographics and lymph nodes distribution of PTC patients with HT and without HT.

Variables	With HT	Without HT	*p* value
Patients	124	109	
Sex			0.002
Male Female	29 95	46 63	
Age (years)			0.002
Median (interquartile range)	32.0 (28.0–40.0)	37.0 (31.0- 44.5)	
LNs distribution Metastatic Nonmetastatic	255 62 193	224 93 131	0.001
Lateral LNs Metastatic Nonmetastatic	174 25 149	166 35 131	0.118

Note: PTC, papillary thyroid cancer; HT, Hashimoto’s thyroiditis; LN, lymph nodes

*p* values indicate the comparisons among sex, age, and numbers of metastatic and nonmetastatic LNs in the cervical and lateral cervical regions between patients with HT and without HT in column order

### DECT examination

Imaging acquisition was performed using a third-generation dual-source DECT scanner (Somatom Force; Siemens Healthcare). Scans ranged from the skull base to the upper margin of the aortic arch. Detailed scan parameters were as follows: tube A voltage, 80 kV; tube B voltage, Sn150 kV; detector configuration, 128 × 0.6 mm; rotation time, 0.5 s; pitch, 0.7; and matrix, 256 × 256. Automatic attenuation-based tube current modulation (CARE Dose 4D; Siemens Healthcare) combined with an iterative reconstruction algorithm (ADMIRE, Siemens Healthcare, Strength Level 3) was used to reduce the radiation dose. For contrast-enhanced scan, 75 mL of contrast agent (iopromide; Bayer Healthcare) was injected into the elbow vein at a flow of 3.5 mL/s using an automated high-pressure syringe. Image acquisition of the arterial phase (AP) and venous phase (VP) started at 25 and 50 s after contrast agent injection, respectively.

### LN histopathologic marking on DECT images

The LN levels were recorded according to the American Joint Committee on Cancer cervical regional lymph node level system. The status of LNs was defined according to surgical pathology reports of neck dissection. LN histopathologic marking on DECT images was in accordance with one method proposed in a previous study [17]. If all the LNs in one level were histologically proven to be metastatic, then the LNs visible on DECT images at this level were all assigned as metastatic LNs. If all the LNs in one level were histologically proven to be nonmetastatic, then the LNs visible on DECT images at this level were all assigned as nonmetastatic LNs. If one level contained both metastatic and nonmetastatic LNs, then all the LNs visible on DECT images at this level were excluded from further analyses.

Finally, a total of 479 cervical LNs were enrolled and divided into four groups (1) HT+/LN + group (metastatic LNs in PTC patients with HT, *n* = 63), (2) HT+/LN − group (nonmetastatic LNs in PTC patients with HT, *n* = 193), (3) HT−/LN + group (metastatic LNs in PTC patients without HT, *n* = 93), and (4) HT−/LN − group (nonmetastatic LNs in PTC patients without HT, *n* = 131).

### Image analysis

Iodine maps, Z_eff_ maps, and virtual monochromatic images were reconstructed using commercially available software (*syngo*.via, version VB10B; Siemens Healthcare). A circular region of interest was drawn manually on the axial slice showing the maximum short axis diameter of LN, avoiding vessels and necrotic areas. After the region of interest was placed, IC and Z_eff_ could be obtained automatically. Normalized IC was calculated as follows: $IC = I{C_{LN}}/I{C_{CCA}}$, where IC_LN_ is the IC of LN and IC_CCA_ is the IC of the common carotid artery at the same level as the LN. Energy spectrum curves at 40 to 190 keV energy levels were plotted. The slope of the Hounsfield unit curve (λ_HU_) was calculated as follows: $[{\lambda _{HU}} = (40\,ke{V_{HU}} - 70\,ke{V_{HU}})/30\,\,keV,$, where 40 keV_HU_ and 70 keV_HU_ are the HU values of the 40- and 70-keV monochromatic images, respectively [9]. Measurements were repeated two times, and the average values were calculated for subsequent statistical analyses.

### Statistical analyses

The Kolmogorov–Smirnov test was used to evaluate whether the data were normally distributed or not. Because of the non-normal distribution of the parameters in the four groups, all data were presented as median (interquartile range). The Kruskal–Wallis test with subsequent post-hoc pairwise was then used to compare the differences of continuous variables among the four groups. Receiver operating characteristic curves together with the area under the curve (AUC), sensitivity and specificity were used to evaluate the diagnostic performance and threshold of DECT-derived quantitative parameters. Statistical analyses were performed using SPSS software (version 26.0; IBM). All *p* values were two-sided with significance at less than 0.05.

## Results

### Effect of HT on DECT-derived quantitative parameters of cervical metastatic and nonmetastatic LNs in PTC patients

**Fig. 1:**
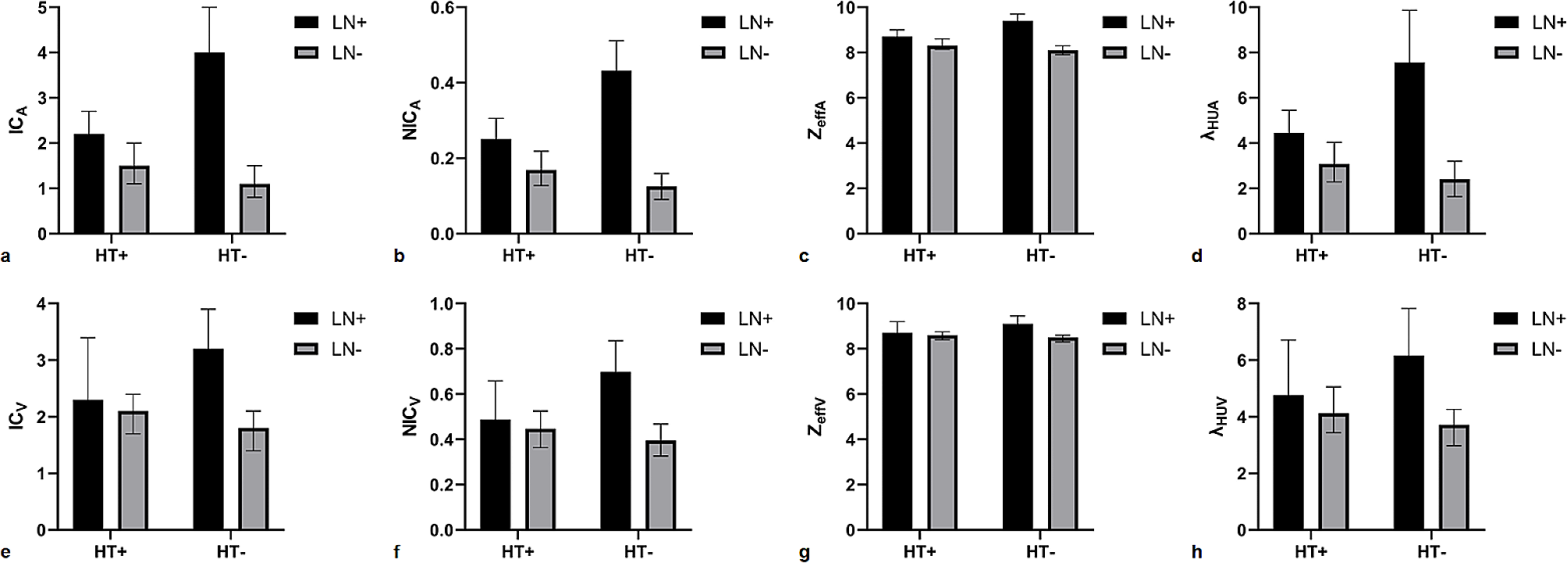
IC_A_/IC_v_ (**a**, **e**), NIC_A_/NIC_V_ (**b**, **f**), Z_effA_/Z_effV_ (**c**, **g**), and λ_HUA_/λ_HUV_ (**d**, **h**) of metastatic and nonmetastatic lymph nodes in PTC patients with HT and without HT. All *p* values except NIC_V_ between LN + and LN − in HT + patients are greater than 0.05. PTC, papillary thyroid cancer; HT, Hashimoto’s thyroiditis; HT+, PTC with HT; HT−, PTC without HT; LN+, metastatic lymph nodes; LN−, nonmetastatic LNs; IC_A_/IC_v_, iodine concentration in arterial/venous phase; NIC_A_/NIC_V_, normalized IC_A_/IC_v_; Z_effA_/Z_effV,_ effective atomic number in arterial/venous phase; and λ_HUA_/λ_HUV_, slope of the spectral Hounsfield unit curve in arterial/venous phase

**Fig. 2:**
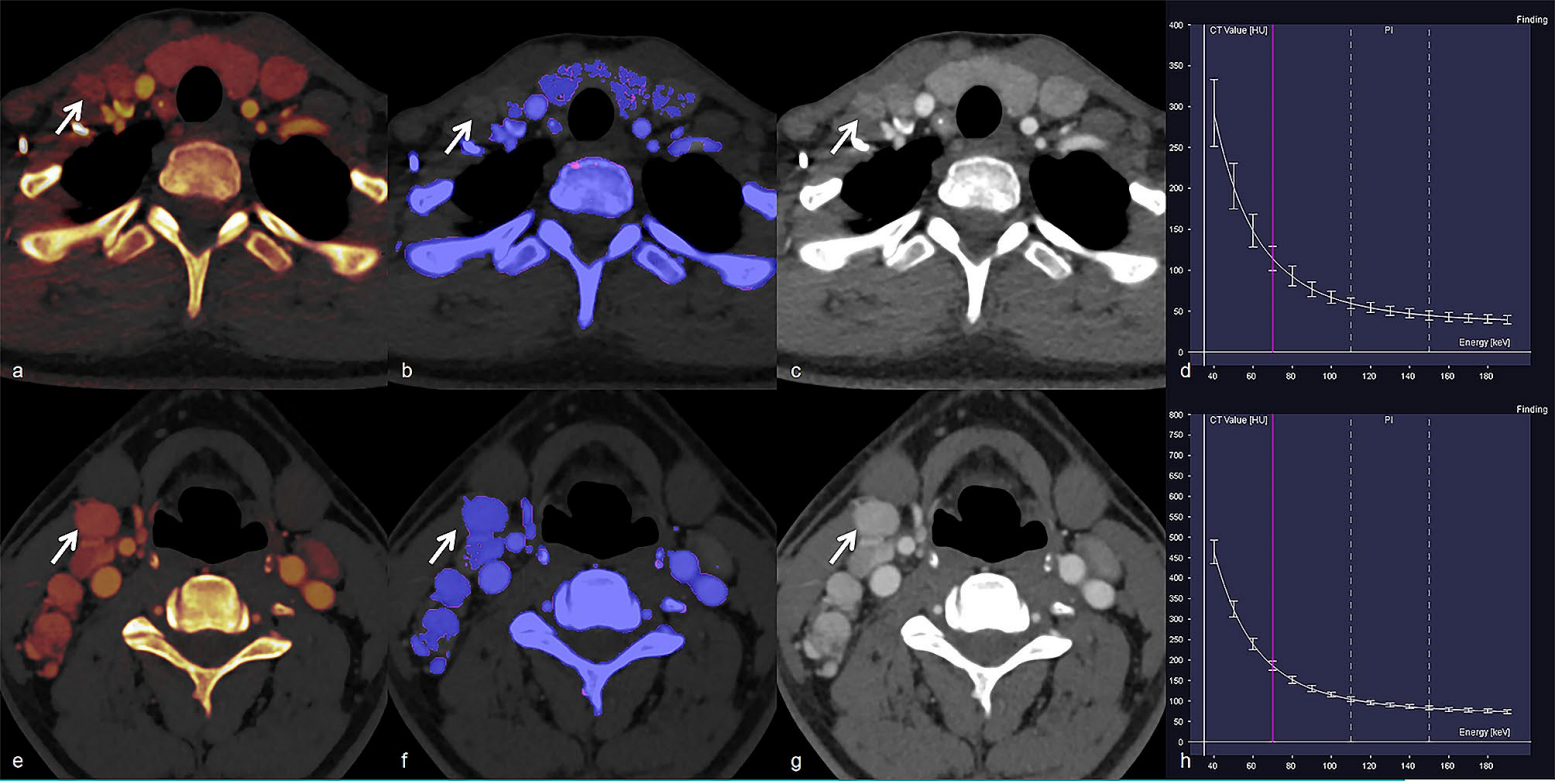
Representative images and energy spectrum curves of metastatic lateral lymph nodes in PTC patients with HT (**a**–**d**) and without HT (**e–h**). Metastatic LNs in PTC patients with HT demonstrated lower IC (3.000 vs. 6.300 mg/mL), NIC (0.248 vs. 0.670), Z_eff_ (9.000 vs. 10.200), and λ_HU_ (5.924 vs. 11.313) than those in PTC patients without HT in arterial phase. PTC, papillary thyroid cancer; HT, Hashimoto’s thyroiditis; LN, lymph node; IC, iodine concentration; NIC, normalized IC; Z_eff_, effective atomic number; and λ_HU_, slope of the spectral Hounsfield unit curve

**Fig. 3:**
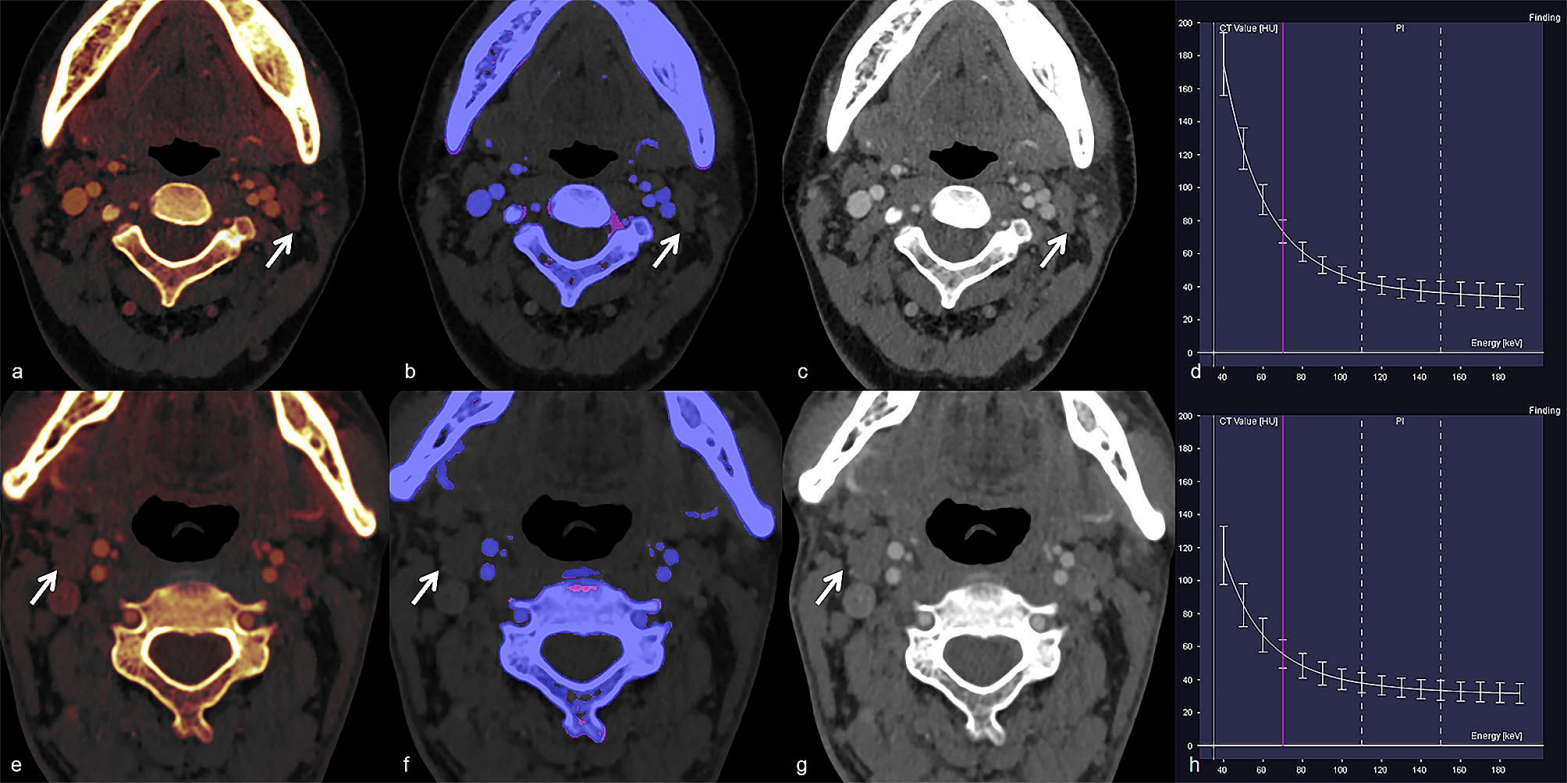
Representative images and energy spectrum curves of nonmetastatic lateral lymph nodes in PTC patients with HT (**a**–**d**) and without HT (**e–h**). Nonmetastatic LNs in PTC patient with HT demonstrated higher IC (1.700 vs. 0.900 mg/mL), NIC (0.210 vs. 0.134), Z_eff_ (8.400 vs. 8.000), and λ_HU_ (3.381 vs. 1.989) than those in PTC patients without HT in arterial phase. PTC, papillary thyroid cancer; HT, Hashimoto’s thyroiditis; LN, lymph node; IC, iodine concentration; NIC, normalized IC; Z_eff_, effective atomic number; and λ_HU_, slope of the spectral Hounsfield unit curve

DECT quantitative parameters of the four groups are summarized in Table 2 (Fig. 1a–h). Significant differences were observed in all DECT quantitative parameters in both the AP and VP among the four groups (all *p* < 0.001; Table 2). The values of all DECT quantitative parameters measured in the HT+/LN + group were significantly lower than those in the HT−/LN + group, whereas they were significantly higher in the HT+/LN − group than in the HT−/LN − group (all *p* < 0.05; Table 2). Representative cases are shown in Figs. 2 and 3.

**Table 2:** Comparison of quantitative dual-energy CT parameters between metastatic and nonmetastatic lymph nodes in PTC patients with and without HT

	HT+/LN+ (*n* = 62)	HT+/LN- (*n* = 193)	HT-/LN+ (*n* = 93)	HT-/LN- (*n* = 131)	*p* value	*p*^a^ value	*p*^b^ value	*p*^c^ value	*p*^d^ value
Arterial phase									
IC (mg/mL)	2.200 (1.600–2.700)	1.500 (1.100–2.000)	4.000 (3.000–5.000)	1.100 (0.800–1.500)	< 0.001	< 0.001	< 0.001	< 0.001	< 0.001
NIC	0.251 (0.193–0.306)	0.169 (0.128–0.219)	0.432 (0.335–0.512)	0.125 (0.091–0.160)	< 0.001	< 0.001	< 0.001	< 0.001	< 0.001
Z_eff_	8.700 (8.400–9.000)	8.300 (8.150–8.600)	9.400 (9.050–9.700)	8.100 (7.900–8.300)	< 0.001	< 0.001	< 0.001	< 0.001	< 0.001
λ_HU_	4.454 (3.515–5.457)	3.086 (2.289–4.041)	7.573 (5.518–9.867)	2.408 (1.641–3.204)	< 0.001	< 0.001	< 0.001	< 0.001	< 0.001
Venous phase									
IC (mg/mL)	2.300 (1.775–3.400)	2.100 (1.700–2.400)	3.200 (2.600–3.900)	1.800 (1.400–2.100)	< 0.001	0.018	< 0.001	< 0.001	0.001
NIC	0.488 (0.385–0.658)	0.447 (0.364–0.525)	0.698 (0.570–0.837)	0.395 (0.326–0.468)	< 0.001	0.167	< 0.001	< 0.001	0.004
Z_eff_	8.700 (8.400–9.200)	8.600 (8.400–8.750)	9.100 (8.800–9.450)	8.500 (8.300–8.600)	< 0.001	0.045	< 0.001	< 0.001	0.001
λ_HU_	4.760 (3.856–6.708)	4.121 (3.434–5.056)	6.166 (4.795–7.828)	3.718 (2.979–4.264)	< 0.001	0.005	< 0.001	0.003	0.001

Note: PTC, papillary thyroid cancer; HT, Hashimoto’s thyroiditis; HT+, PTC with HT; HT-, PTC without HT; LN+, metastatic lymph nodes; LN-, nonmetastatic LNs; IC, iodine concentration; NIC, normalized IC; Z_eff_, effective atomic number; λ_HU_, slope of the spectral Hounsfield unit curve

*p*^a^ value, HT+/LN + vs. HT+/LN-; *p*^b^ value, HT-/LN + vs. HT-/LN-; *p*^c^ value, HT+/LN + vs. HT-/LN+; *p*^d^ value, HT+/LN- vs. HT-/LN-.

### Effect of HT on the performance or threshold of DECT in preoperatively diagnosing metastatic cervical LNs in PTC patients

The HT+/LN + group exhibited significantly higher NIC in the AP and higher IC, Z_eff_ and λ_HU_ in both the AP and VP than the HT+/LN − group (all *p* < 0.05; Table 2). The values of all DECT parameters measured in both the AP and VP were significantly higher in the HT−/LN + group than those in the HT−/LN− group (all *p* < 0.001; Table 2).

**Fig. 4:**
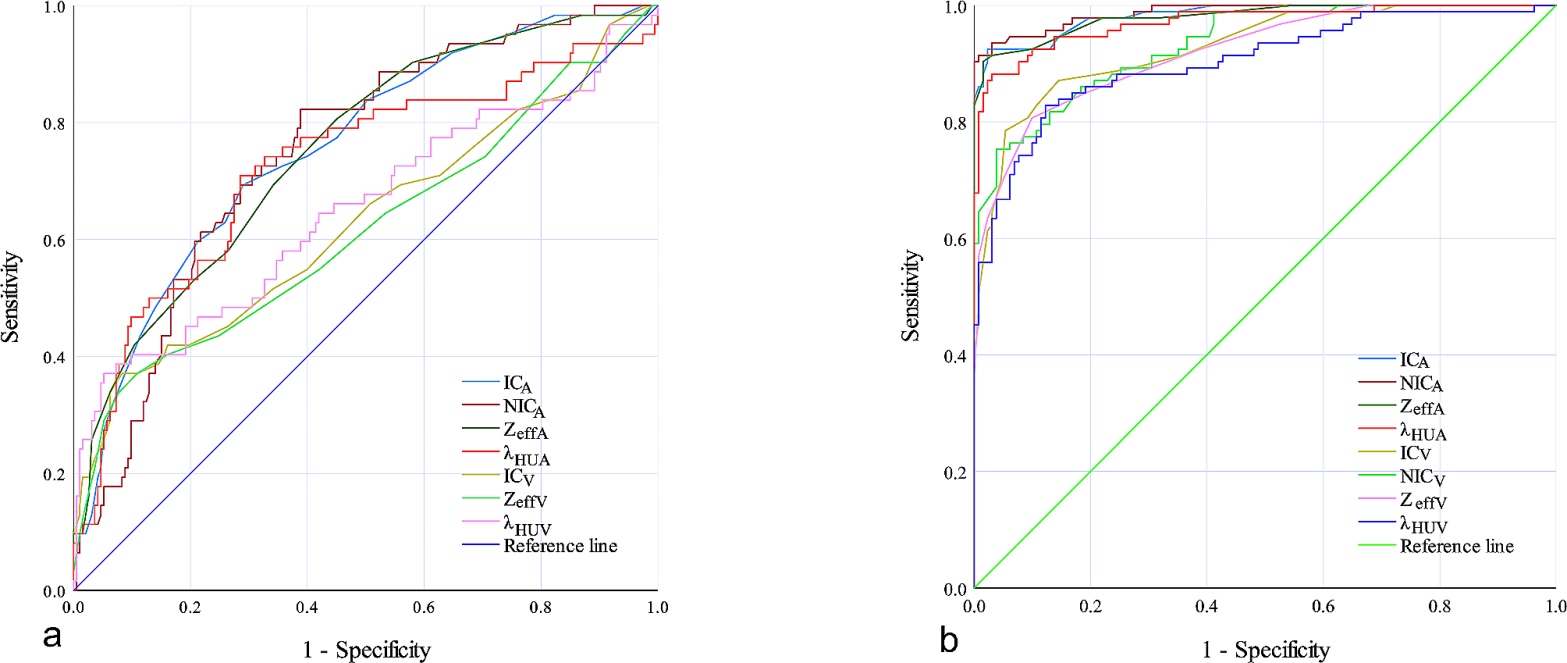
ROC curves using IC_A_/IC_v_, NIC_A_/NIC_V_, Z_effA_/Z_effV_ and λ_HUA_/λ_HUV_ in differentiating metastatic and nonmetastatic lymph nodes in PTC patients with HT (**a**) and without HT (**b**). PTC, papillary thyroid cancer; HT, Hashimoto’s thyroiditis; IC_A_/IC_v_, iodine concentration in arterial/venous phase; NIC_A_/NIC_V_, normalized IC_A_/IC_v_; Z_effA_/Z_effV_, effective atomic number in arterial/venous phase; λ_HUA_/λ_HUV_, slope of the spectral Hounsfield unit curve in arterial/venous phase; and ROC, receiver operating characteristic

The diagnostic performance, sensitivity, and specificity of each DECT parameter in differentiating metastatic from nonmetastatic LNs in both the HT + and HT − groups are listed in Table 3. The thresholds of these DECT parameters differed in the HT + and HT − PTC patients, and the AUCs were all greater than 0.600 (Table 3; Fig. 4). In the HT + group, the AUCs of DECT parameters in the AP were greater than 0.700, which indicated higher diagnostic value. In the HT − group, the AUCs of all DECT parameters in the AP and VP were greater than 0.900, which indicated excellent performance.

**Table 3:** Diagnostic performance of dual-energy CT parameters in differentiating metastatic from nonmetastatic lymph nodes in PTC patients with and without HT

	Threshold	AUC	Sensitivity	Specificity
HT+/LN + vs. HT+/LN-				
IC_A_ (mg/mL) NIC_A_ Z_effA_ λ_HUA_ IC_V_ (mg/mL) Z_effV_ λ_HUV_	1.850 0.188 8.350 3.798 2.950 8.950 6.116	0.757 0.749 0.753 0.726 0.630 0.616 0.651	0.694 0.823 0.806 0.710 0.371 0.371 0.371	0.710 0.611 0.549 0.715 0.917 0.891 0.948
HT-/LN + vs. HT-/LN-				
IC_A_ (mg/mL) NIC_A_ Z_effA_ λ_HUA_ IC_V_ (mg/mL) NIC_V_ Z_effV_ λ_HUV_	2.050 0.243 8.650 4.310 2.550 0.576 8.750 4.601	0.983 0.988 0.980 0.971 0.929 0.932 0.920 0.903	0.925 0.914 0.903 0.882 0.785 0.753 0.806 0.828	0.977 0.992 0.985 0.969 0.947 0.962 0.901 0.878

Note: PTC, papillary thyroid cancer; HT, Hashimoto’s thyroiditis; HT+, PTC with HT; HT-, PTC without HT; LN+, metastatic lymph nodes; LN-, nonmetastatic LNs; IC_A_/IC_v_, iodine concentration in arterial/venous phase; NIC_A_/NIC_V_, normalized IC_A_/IC_v_; Z_effA_/Z_effV_, effective atomic number in arterial/venous phase; λ_HUA_/λ_HUV_, slope of the spectral Hounsfield unit curve in arterial/venous phase. AUC, area under the curve

Threshold IC_A_/IC_v_ are represented as mg/mL

In the HT + group, if an AP-IC of 1.850 mg/mL was used as a threshold value, then optimal diagnostic performance (AUC, 0.757; sensitivity, 69.4%; and specificity, 71.0%) could be obtained (Table 3; Fig. 4a). In contrast, in the HT − group, the optimal threshold value of AP-IC was 2.050 mg/mL (Table 3; Fig. 4b).

However, in the HT − group, AP-NIC demonstrated the highest AUC of 0.988, sensitivity of 91.4%, and specificity of 99.2% when an optimal threshold of 0.243 was used (Table 3; Fig. 4b). In contrast, in the HT + group, the optimal threshold value of AP-NIC was 0.188 (Table 3; Fig. 4a).

### Subgroup analysis of the effect of HT on DECT-derived quantitative parameters of lateral cervical metastatic and nonmetastatic LNs

**Fig. 5:**
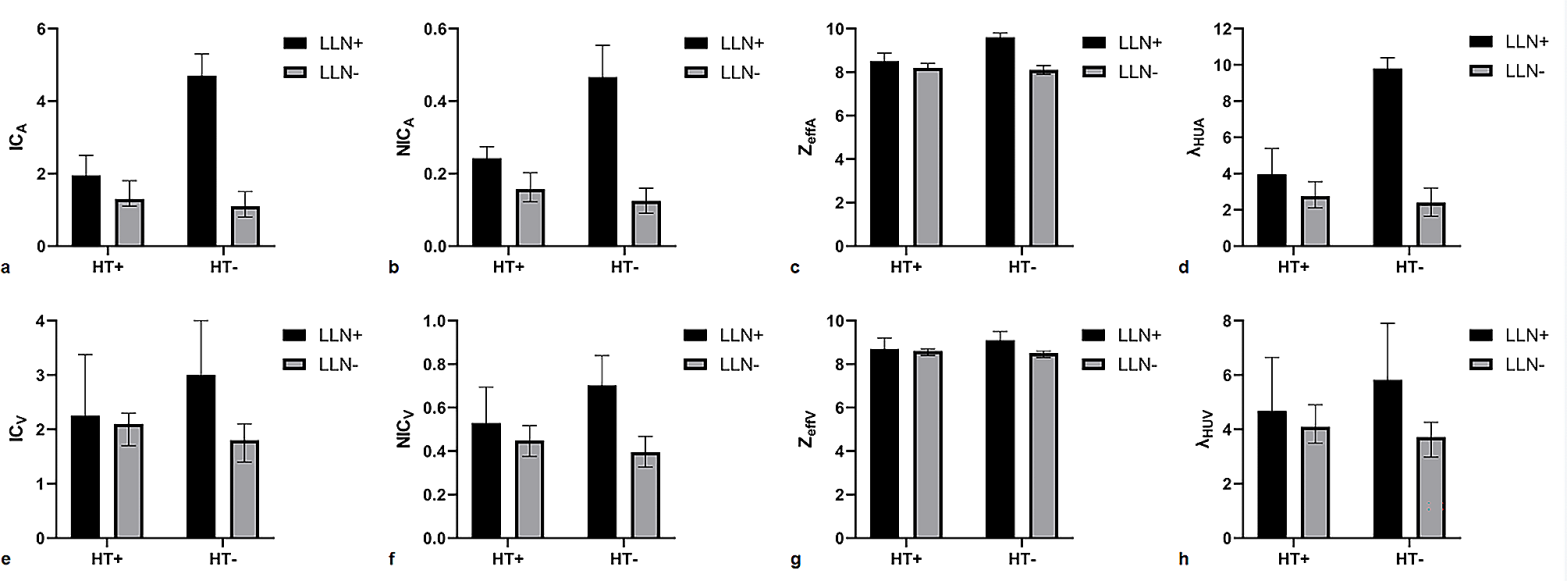
IC_A_/IC_v_ (**a**, **e**), NIC_A_/NIC_V_ (**b**, **f**), Z_effA_/Z_effV_ (**c**, **g**), and λ_HUA_/λ_HUV_ (**d**, **h**) of metastatic and nonmetastatic lymph nodes in PTC patients with HT and without HT. All *p* values except λ_HUV_ between LN + and LN − in HT + patients are greater than 0.05. PTC, papillary thyroid cancer; HT, Hashimoto’s thyroiditis; HT+, PTC with HT; HT−, PTC without HT; LN+, metastatic lymph nodes; LN-, nonmetastatic LNs. IC_A_/IC_v_, iodine concentration in arterial/venous phase; NIC_A_/NIC_V_, normalized IC_A_/IC_v_; Z_effA_/Z_effV,_ effective atomic number in arterial/venous phase; and λ_HUA_/λ_HUV_, slope of the spectral Hounsfield unit curve in arterial/venous phase

We further compared DECT quantitative parameters of lateral LNs among the four groups (Table 4; Fig. 5a–h). Similarly, the values of the DECT quantitative parameters of the lateral HT+/LN + group were significantly lower than those of the lateral HT−/LN + group, whereas the values of the parameters of the lateral HT+/LN − group were significantly higher than those of the parameters of the lateral HT−/LN − group (all *p* < 0.05; Table 4).

**Table 4:** Comparison of quantitative dual-energy CT parameters between lateral metastatic and nonmetastatic lymph nodes in PTC patients with and without HT

	HT+/LLN+ (*n* = 24)	HT+/LLN- (*n* = 149)	HT-/LLN+ (*n* = 35)	HT-/LLN- (*n* = 131)	*p* value	*p*^a^ value	*p*^b^ value	*p*^c^ value	*p*^d^ value
Arterial phase									
IC (mg/mL)	1.950 (1.600–2.500)	1.300 (1.100–1.800)	4.700 (3.500–5.300)	1.100 (0.800–1.500)	< 0.001	< 0.001	< 0.001	0.022	< 0.001
NIC	0.243 (0.193–0.274)	0.157 (0.122–0.202)	0.467 (0.357–0.554)	0.125 (0.091–0.160)	< 0.001	< 0.001	< 0.001	0.021	< 0.001
Z_eff_	8.500 (8.400–8.875)	8.200 (8.100–8.400)	9.600 (9.300–9.800)	8.100 (7.900–8.300)	< 0.001	< 0.001	< 0.001	0.022	< 0.001
λ_HU_	3.968 (3.447–5.390)	2.757 (2.104–3.560)	9.807(6.403–10.395)	2.408 (1.641–3.204)	< 0.001	< 0.001	< 0.001	0.029	0.005
Venous phase									
IC (mg/mL)	2.250 (2.025–3.375)	2.100 (1.700–2.300)	3.000 (2.500–4.000)	1.800 (1.400–2.100)	< 0.001	0.013	< 0.001	0.018	< 0.001
NIC	0.529 (0.412–0.695)	0.449 (0.376–0.517)	0.703 (0.591–0.840)	0.395 (0.326–0.468)	< 0.001	0.025	< 0.001	0.009	< 0.001
Z_eff_	8.700 (8.500–9.200)	8.600 (8.400–8.700)	9.100 (8.800–9.500)	8.500 (8.300–8.600)	< 0.001	0.040	< 0.001	0.007	< 0.001
λ_HU_	4.675 (3.682–6.652)	4.100 (3.489–4.902)	5.826 (4.748–7.902)	3.718 (2.979–4.264)	< 0.001	0.060	< 0.001	0.011	< 0.001

Note: PTC, papillary thyroid cancer; HT, Hashimoto’s thyroiditis; HT+, PTC with HT; HT-, PTC without HT; LLN+, metastatic lateral lymph nodes; LLN-, nonmetastatic LLNs; IC, iodine concentration; NIC, normalized IC; Z_eff_, effective atomic number; λ_HU_, slope of the spectral Hounsfield unit curve

*p*^a^ value, HT+/LLN + vs. HT+/LLN-; *p*^b^ value, HT-/LLN + vs. HT-/LLN-; *p*^c^ value, HT+/LLN + vs. HT-/LLN+; *p*^d^ value, HT+/LLN- vs. HT-/LLN-.

**Subgroup analysis of the effect of HT on the performance of DECT in preoperatively diagnosing lateral cervical metastatic LNs**.

The lateral HT+/LN − group exhibited significantly lower values of all DECT quantitative parameters than the lateral HT+/LN + group (all *p* < 0.05; Table 4), except λ_HU_ in the VP (*p* = 0.060). In addition, the values of all DECT quantitative parameters measured in both the AP and VP of the lateral HT−/LN + group were significantly higher than those of the lateral HT−/LN − group (all *p* < 0.001; Table 4).

**Fig. 6:**
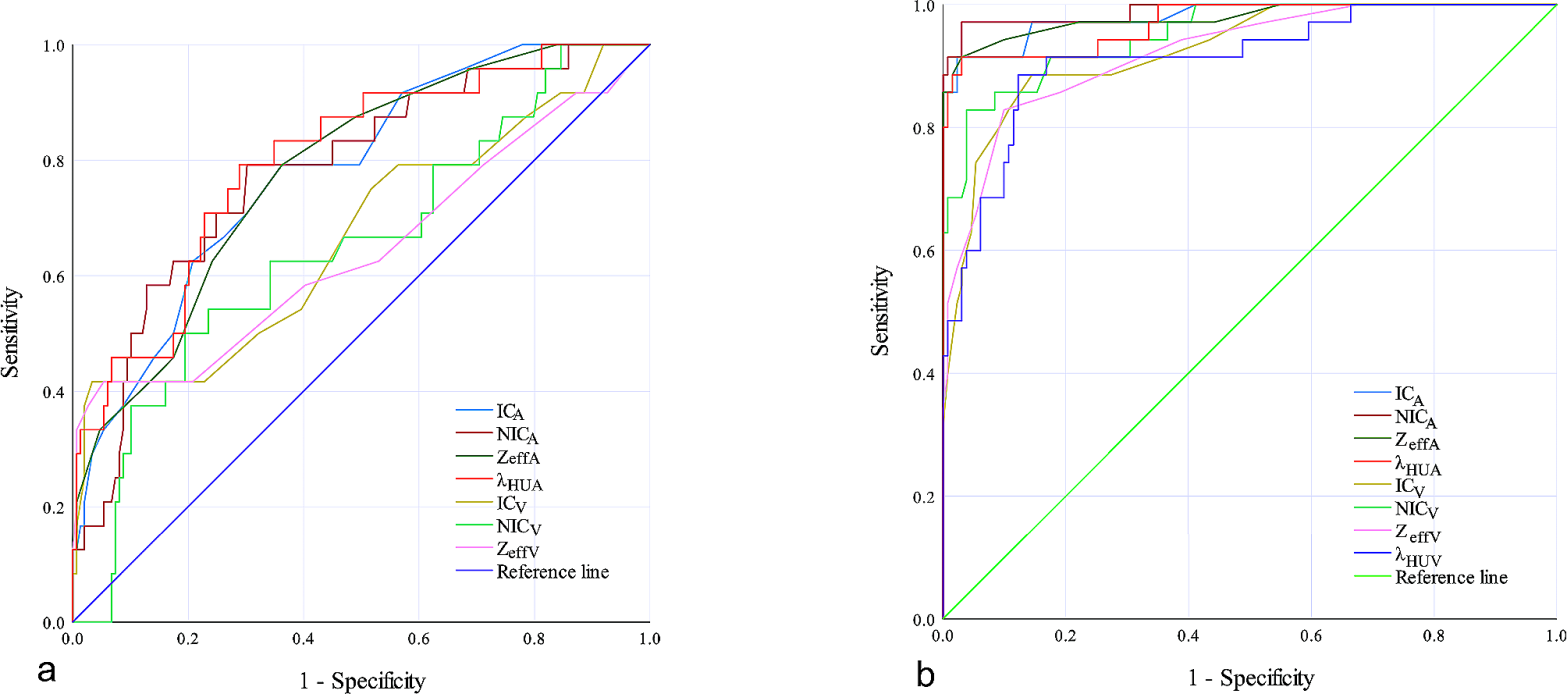
ROC curves using IC_A_/IC_v_, NIC_A_/NIC_V_, Z_effA_/Z_effV_, and λ_HUA_/λ_HUV_ in differentiating lateral metastatic and nonmetastatic lymph nodes in PTC patients with HT (**a**) and without HT (**b**). PTC, papillary thyroid cancer; HT, Hashimoto’s thyroiditis; IC_A_/IC_v_, iodine concentration in arterial/venous phase; NIC_A_/NIC_V_, normalized IC_A_/IC_v_; Z_effA_/Z_effV_, effective atomic number in arterial/venous phase; λ_HUA_/λ_HUV,_ slope of the spectral Hounsfield unit curve in arterial/venous phase; and ROC, receiver operating characteristic

Diagnostic performance, sensitivity and specificity of each DECT quantitative parameter in differentiating lateral metastatic from nonmetastatic LNs in both the HT + and HT − groups are presented in Table 5. The thresholds of these DECT parameters differed between the HT + and HT- patients, with all AUCs greater than 0.600 (Table 5; Fig. 6). In the HT + group, the AUCs of DECT parameters in the AP were greater than 0.700, indicating higher diagnostic ability. In the HT − group, all DECT parameters in the AP and VP indicated excellent performance with AUCs greater than 0.900.

**Table 5:** Diagnostic performance of dual-energy CT parameters in differentiating lateral metastatic from nonmetastatic lymph nodes in PTC patients with and without HT

	Threshold	AUC	Sensitivity	Specificity
HT+/LLN + vs. HT+/LLN-				
IC_A_ (mg/mL) NIC_A_ Z_effA_ λ_HUA_ IC_V_ (mg/mL) NIC_V_ Z_effV_	1.550 0.193 8.350 3.398 2.950 0.523 8.950	0.780 0.780 0.780 0.800 0.668 0.649 0.644	0.792 0.792 0.792 0.792 0.417 0.542 0.417	0.638 0.698 0.638 0.711 0.966 0.765 0.946
HT-/LLN + vs. HT-/LLN-				
IC_A_ (mg/mL) NIC_A_ Z_effA_ λ_HUA_ IC_V_ (mg/mL) NIC_V_ Z_effV_ λ_HUV_	2.050 0.226 8.550 4.310 2.250 0.577 8.750 4.601	0.980 0.989 0.978 0.971 0.926 0.952 0.922 0.914	0.914 0.971 0.914 0.914 0.886 0.829 0.829 0.886	0.977 0.969 0.969 0.969 0.855 0.962 0.901 0.878

Note: PTC, papillary thyroid cancer; HT, Hashimoto’s thyroiditis; HT+, PTC with HT; HT-, PTC without HT; LLN+, metastatic lateral lymph nodes; LLN-, nonmetastatic LLNs; IC_A_/IC_v_, iodine concentration in arterial/venous phase; NIC_A_/NIC_V_, normalized IC_A_/IC_v_; λ_HUA_/λ_HUV_, slope of the spectral Hounsfield unit curve in arterial/venous phase; Z_effA_/Z_effV_, effective atomic number in arterial/venous phase. AUC, area under the curve

Threshold IC_A_/IC_v_ are represented as mg/mL

Receiver operating characteristic curve analyses demonstrated that the AP-λ_HU_ had optimal diagnostic performance (AUC, 0.800; sensitivity, 79.2%; specificity, 77.1%) for diagnosing lateral metastatic LNs in the HT + group when 3.398 was used as the threshold value (Table 5; Fig. 6a). In contrast, in the HT − PTC group, the optimal threshold value of AP-λ_HU_ was 4.310 (Table 5; Fig. 6b).

However, in the HT − group, AP-NIC showed optimal diagnostic performance (AUC, 0.989; sensitivity, 97.1%; and specificity, 96.9%) when 0.226 was used as the threshold value (Table 5; Fig. 6b). In contrast, in the HT + group, the optimal threshold of AP-NIC was 0.193 (Table 5; Fig. 6a).

## Discussion

In the present study, we observed that the coexistence of HT may influence the DECT quantitative parameters of cervical LNs. Metastatic LNs in the HT + group exhibited lower values of DECT quantitative parameters than those in the HT − group, whereas nonmetastatic LNs in the HT + group exhibited higher values of DECT quantitative parameters. Furthermore, the optimal threshold values of DECT quantitative parameters for diagnosing metastatic cervical LNs differed between the HT + and HT − groups. These findings remained consistent in the subgroup analysis of lateral cervical LNs. To the best of our knowledge, our study was the first to evaluate the effect of HT on DECT quantitative parameters themselves, their performance and thresholds in preoperatively diagnosing metastatic cervical LNs in patients with PTC.

HT is an autoimmune disease characterized by lymphocyte and macrophage infiltration within and surrounding the tumors, which could interfere with the tumor microenvironment to improve antitumor immune responses [18, 19]. The presence of inflammatory and immune responses may be combined to result in a lower rate of angiolymphatic invasion in the HT + group [16]. As a result, metastatic LNs in the HT + group exhibited lower values of DECT quantitative parameters than those in the HT − group. In addition, previous studies have reported that the clonal expansion and maturation of autoreactive CD4 + T cells, CD8 + cytotoxic T cells, and B cells could result in the uncontrolled production of autoantibodies in the central stage of the development of HT, subsequently leading to cervical lymphadenopathy. This situation can increase the misdiagnosis risks of metastatic LNs based on imaging [14, 20]. This may be ascribed to the increased vessel density in reactive inflammatory nonmetastatic LNs in the HT + group [21]. Thus, they would present higher IC, NIC, Z_eff_ and λ_HU_ than nonmetastatic LNs in the HT − group. US is the most common method used for assessing the status of LNs in patients with PTC. The effect of HT on the diagnostic efficacy of US for cervical LNs in PTC patients has also been investigated in a previous study [22]. Tan HL et al. reported that HT can interfere with the evaluation of US by increasing the frequency of fatty hilum absence in benign central LNs, thereby reducing its diagnostic accuracy and increasing the misdiagnosis rate [22]. Therefore, according to our findings, we believe that the LNs of PTC patients with concomitant HT should be thoroughly evaluated by clinicians, wherein DECT could serve as an indispensable adjunct modality.

IC and NIC provide an objective and quantitative assessment of iodine contrast agent uptake, which indirectly reflects the angiogenesis of lesions [23, 24]. Z_eff_ provides information on the elemental composition of materials, whereas λ_HU_ is determined by the physical and chemical nature of tissues [25]. The alterations in IC and NIC will cause differences in the composition and characteristics of tissues, leading to changes in Z_eff_ and λ_HU_. Our study revealed again that the values of the DECT parameters of the metastatic LNs in both the arterial and venous phases were significantly higher than those of the nonmetastatic LNs, which was in line with the previous studies [9, 26]. This finding may be ascribed to the abundant blood supply in metastatic LNs [27]. However, because of the effect of HT on the DECT parameters themselves, the diagnostic performance and thresholds differed in the HT + and HT − groups. The findings of the present study suggested that whether HT was coexistent or not should be clarified before imaging diagnosis and that it is crucial during the interpretation of imaging features and parameters.

Lateral LN metastasis was considered as a risk factor for disease recurrence and poor prognosis of PTC [28]. Lateral neck dissection is widely considered a necessary procedure for patients with confirmed lateral metastatic LNs [2]. Therefore, preoperative identification of lateral LN metastasis is essential for PTC patients. Our subgroup analysis of lateral LN confirmed again the potential influence of HT on DECT parameters themselves, their performance, and thresholds and emphasized the importance of clarifying whether HT was coexistent or not beforehand.

There were several limitations in our study. First, this was a retrospective single-center study with inevitable selection bias. Second, the pathological findings of the HT + and HT − LNs were not studied. Further study corresponding to DECT and pathological findings would be valuable to confirm and explain our results.

## Conclusions

Our findings indicated that the presence of coexistent HT affected the DECT quantitative parameters of cervical LNs in patients with PTC. Furthermore, the optimal thresholds of DECT quantitative parameters for diagnosing metastatic LNs in the HT + and HT − groups may be different. Before diagnosing the metastatic LNs in patients with PTC with reference to the DECT quantitative parameters, whether HT was coexistent should be clarified.

## Data Availability

The datasets used and/or analysed during the current study are available from the corresponding author on reasonable request.

## Abbreviations

PTC: Papillary thyroid cancer
LN: Lymph node
US: Ultrasonography
CT: Computed tomography
DECT: Dual-energy computed tomography
IC: Iodine concentration
NIC: Normalized iodine concentration
Z_eff_: Effective atomic number
λ_HU_: Slope of the spectral Hounsfield unit curve
AP: Arterial phase
VP: Venous phase
HT: Hashimoto’s thyroiditis
AUC: Area under the curve
